# Cell metabolomics study on the anticancer effects of *Ophiopogon japonicus* against lung cancer cells using UHPLC/Q-TOF-MS analysis

**DOI:** 10.3389/fphar.2022.1017830

**Published:** 2022-09-16

**Authors:** Qiao Liu, Jia-Man Shen, Hui-Jie Hong, Qi Yang, Wen Liu, Zhong Guan, Yi-Tao Wang, Xiao-Jia Chen

**Affiliations:** ^1^ State Key Laboratory of Characteristic Chinese Medicine Resources in Southwest China, College of Pharmacy, Chengdu University of Traditional Chinese Medicine, Chengdu, China; ^2^ Institute of Chinese Medical Sciences, State Key Laboratory of Quality Research in Chinese Medicine, University of Macau, Taipa, Macao SAR, China; ^3^ Zhuhai UM Science and Technology Research Institute, Zhuhai, China

**Keywords:** *Ophiopogon japonicus*, lung cancer, cell metabolomics, UHPLC/Q-TOF-MS, glycerophospholipid metabolism, ether lipid metabolism, glutathione metabolism

## Abstract

*Ophiopogon japonicus* (OJ) is a traditional Chinese herbal medicine that has been used for thousands of years. Recently, the anticancer effects of OJ have been reported in multiple types of cancer, particularly in lung cancer. However, the underlying mechanisms remain unclear. In present study, the effects of OJ against NCI-H1299 human lung cancer cells were investigated, and the underlying mechanisms were explored using ultra-high-performance liquid chromatography-quadrupole time-of-flight mass spectrometry (UHPLC/Q-TOF-MS)-based cell metabolomics. As a result, OJ inhibited the proliferation, induced the apoptosis and suppressed the migration of NCI-H1299 cells. A total of 22 differential metabolites responsible for the effects of OJ were screened and annotated based on the LC-MS-based cell metabolomics approach. The altered metabolites were involved in three metabolic pathways, including glycerophospholipid metabolism, ether lipid metabolism and glutathione metabolism. These results showed that cell metabolomics-based strategies are promising tools to discover the action mechanisms of OJ against lung cancer cells.

## 1 Introduction

*Ophiopogon japonicus* (Thunb.) Ker Gawl. (OJ), is a well-known Chinese herbal medicine that has been used for thousands of years (Chen M.-H. et al., 2016). OJ is commonly used to improve immunity and nourish the lungs, thereby relieving cough as recorded in the Chinese pharmacopoeia (2020 edition). Modern research has shown that OJ contains steroidal saponins, homoisoflavonoids, polysaccharides, organic acids, volatile oils and trace elements, which possess the pharmacological effects of cardiovascular protection, anti-diabetes, anti-inflammation, antioxidant, anticancer, anti-aging and immunomodulation (Kou et al., 2005; Li et al., 2012; Bi et al., 2013; Fan et al., 2015; Zhang J. et al., 2016; Zhao J. W. et al., 2017; Chen et al., 2017; Ercan et al., 2020). OJ is mainly produced in Sichuan and Zhejiang provinces in China, which is called Zhejiang OJ (ZOJ) and Sichuan OJ (COJ), respectively (Zhao M. et al., 2017). Previous reports show that OJ from the two regions contain similar types of steroidal saponins and homoisoflavonoids, but their composition and concentration are different. ZOJ contains higher contents of homoisoflavonoids than COJ, while COJ and ZOJ show distinctive composition of saponins (Lin et al., 2010; Zhao M. et al., 2017). Differences in the chemical composition may lead to different pharmacological effects of OJ from different regions.

Recent reports showed that OJ can inhibit tumor growth, induce apoptosis and suppress metastasis in various types of cancers *in vitro* and *in vivo* (Zang et al., 2016; Chen et al., 2017), particularly in lung cancer. For instance, the steroidal saponins of OJ and flavonoids of OJ significantly inhibit the proliferation of A549 cells (Chen et al., 2017). Ophiopogonin B, a saponin of OJ, inhibits the tumor growth and metastasis in A549-xenografted nude mice (Chen M. et al., 2016; Chen M. et al., 2018). Ophiopogonin D, another saponin of OJ, inhibits tumor growth by inducing apoptosis on non-small cell lung carcinoma model (Lee et al., 2018). However, the underlying mechanisms of the anticancer effects of OJ remain unclear.

Metabolomics is an omics technique that focuses on the study of small molecules and is a high-throughput technique for evaluating large-scale metabolites (Johnson et al., 2016). It uses modern bioanalytical techniques such as nuclear magnetic resonance (NMR) and chromatography-mass spectrometry (MS) to quantify endogenous small molecular metabolites in organisms influenced by internal and external factors such as disease invasion, drug intervention, and environmental factors (Nicholson et al., 1999; Clayton et al., 2006; Shyur and Yang, 2008; Zhang A. et al., 2016; Zhang et al., 2017). Metabolomics emphasizes holism and takes the overall metabolites changes as the research point. It is similar to the theory of traditional Chinese medicines (TCM) “multi-component, multi-target, overall regulation”. Therefore, using metabolomic methods to explore the changes of metabolites under TCM intervention can provide clues to the action mechanism of TCM (Luo et al., 2019; Hou et al., 2021; Pan et al., 2021; Li et al., 2022). In this study, the anticancer effects of OJ against NCI-H1299 cells were evaluated. Subsequently, ultra-high-performance liquid chromatography-quadrupole time-of-flight mass spectrometry (UHPLC/Q-TOF-MS)-based cell metabolomics was utilized to explore the potential metabolite biomarkers and pathways responsible for the anticancer effects of OJ.

## 2 Materials and methods

### 2.1 Chemicals and reagents

Ophiopogonin C, lirioprolioside J and 6- aldehydoisoophiopogonanone A were purchased from Chengdu Desite Biotechnology Co. (Chengdu, China). Ophiopogonin D, ophiopogonin D’, ophiopogonin Ra, methylophiopogonone A, methylophiopogonanone A, methylophiopogonanone B were obtained from Chengdu MUST Bio-technology Co., (Chengdu, China). 14α-Hydroxy-sprengerinin C was purchased from TAUTO biotech Co. (Shanghai, China). Roswell Park Memorial Institute (RPMI) 1640 medium, fetal bovine serum (FBS), phosphate-buffered saline (PBS), trypsin-EDTA and penicillin-streptomycin were bought from Gibco (Carlsbad, CA, United States). Paclitaxel (PTX), 3-(4,5-dimethyl-2-thiazolyl)-2,5-diphenyl-2H-tetrazolium bromide (MTT) and dimethyl sulfoxide (DMSO) were acquired from Sigma Co. Ltd. (St. Louis, MO, United States). Annexin V-FITC Apoptosis Kit was bought from BioVision, Inc. (Milpitas, CA, United States). HPLC-grade methanol, acetonitrile and formic acid were bought from Merck KGaA (Darmstadt, Germany). Ultrapure water was purified by the Millipore purification Milli-Q system (Waters Co. Ltd., Milford, MA, United States) and used for all experiments.

### 2.2 Herbal materials and extraction

OJ samples were collected from Sichuan and Zhejiang provinces of China, respectively. The species of the above samples were identified by Dr. Xiao-Jia Chen, Institute of Chinese Medical Sciences, University of Macau, Macau SAR, China. The OJ ethanol extract (OJE) was prepared as follows: One hundred grams of COJ and ZOJ samples were ground to fine powders, and extracted, respectively, with 1000 ml of ethanol by reflux at 80°C for three times; each extraction lasted for 2 h. Then the extracts were filtered and concentrated in a rotary vacuum evaporator. Finally, the concentrates were lyophilized using a vacuum freeze-dryer, which yielded 6.8 g of COJ ethanol extract (COJE) and 8.5 g ZOJ ethanol extract (ZOJE), respectively.

### 2.3 Chemical analysis of OJE samples

The OJE samples were analyzed by UHPLC-charged aerosol detector (CAD), and 10 components including ophiopogonin C, ophiopogonin Ra, 14α-hydroxy-sprengerinin C, ophiopogonin D, lirioprolioside J, ophiopogonin D’, methylophiopogonone A, methylophiopogonanone A, methylophiopogonanone B, and 6-aldehydoisoophiopogonanone A, were quantitatively determined. The chromatographic analysis was conducted using a Thermo Scientific Ultimate 3000 chromatography system (Thermo Fisher Scientific Inc., Waltham, MA, United States) equipped with a binary gradient pump, an autosampler and a Dionex Corona Veo RS charged aerosol detector (Dionex, Germering, Germany). The chromatographic separations were performed on a Waters ACQUITY UPLC HSS T3 column (2.1 mm × 100 mm, 1.7 μm), equipped with an ACQUITY UPLC HSS T3, VanGuard Pre-Column (Waters, Milford, MA, United States). The mobile phase was composed of water (A) and acetonitrile (B) at a flow rate of 0.4 ml/min. The gradient elution program was: 0–2 min, 30% B, 2–4 min, 30%–35% B; 4–10 min, 35%–40% B; 10–22 min, 40%–43% B; 22–24 min, 43%–46% B; 24–27 min, 46% B; 27–29 min, 46%–49% B; 29–39 min, 49%–52% B; 39–43 min, 52%–100% B; 43–53 min, 100% B; 53–54 min, 100%–30% B; 54–55 min, 30% B. The parameters of CAD were: N_2_ gas pressure, 64.2 psi; evaporator temperature, 35°C; data collection rate, 10 Hz; gain, 8. The injection volume of each sample and standard solution was 5 μl.

### 2.4 Cell culture

The NCI-H1299 and A549 human lung cancer cell lines were obtained from the American Type Culture Collection (ATCC, Rockville, MD, United States). Cells were cultured in RPMI 1640 medium supplemented with 10% FBS and 1% penicillin–streptomycin. All cells were incubated under 5% CO_2_ at 37°C.

### 2.5 Cell proliferation assay and cell morphology

The cell viability of OJE-treated lung cancer cells was estimated by MTT assay. Briefly, NCI-H1299 and A549 cells were seeded at a density of 3,000–5,000 cells/well in 100 μl/well of 96-well plates and were allowed to grow overnight, respectively. OJE and the positive control drug PTX were dissolved in DMSO at the concentrations of 300 mg/ml and 50 mmol/L, respectively. Then the solutions were diluted in culture medium to obtain gradient concentrations. Subsequently, the cells were treated with vehicle (DMSO) or different concentrations of COJE or ZOJE (31.25, 62.5, 125, 250, 500 and 1000 μg/ml) respectively. PTX was administered at the concentrations of 31.25, 62.5, 125, 250, 500 and 1000 nmol/L. After incubation for 24, 48 and 72 h respectively, 10 μl of MTT solution (5 mg/ml) were added in each well and incubated for another 4 h. Subsequently, 150 μl/well of DMSO was added after removing the supernatant to dissolve the purple-colored formazan precipitates. The absorbance at 570 nm was recorded using Flex Station III Multi-Mode Microplate Reader (Molecular Devices, San Jose, California, United States). Cell proliferation (% of control) was calculated by comparing to the OD values of control group. All assays were conducted at least three replicates and repeated three times. Similarly, the cells morphologic alterations after OJE treatment for 48 h were observed with an Incucyte S3 Live-Cell Analysis System (Essen BioScience, Ann Arbor, Michigan, United States).

### 2.6 Cell apoptosis assay

Annexin V-FITC Apoptosis Kit was applied to detect the early apoptosis and late stage of apoptosis or necrosis in OJ treated NCI-H1299 cells. Cells were seeded into 6-well plates at a density of 5.0 × 10^5^ cells/well overnight and then treated with vehicle, various concentrations of OJE (200, or 400 μg/ml) or PTX (500 nmol/L) for 48 h. Cells were washed twice with pre-chilled PBS and collected in 100 μl of 1X binding buffer in tubes. Then, 5 μl PE Annexin V and 5 μl PI were added to the cell buffer, mixed gently and stained at room temperature in the dark for 20 min. Before loading, another 400 μl of 1X binding buffer was added to each sample tube. BC-Cytoflex (Beckman Coulter) was used to analyze the cell death type.

### 2.7 Cell migration assay

The effect of OJE on NCI-H1299 cell migration was assessed using a Transwell chamber (Corning Costar, Lowell, MA). In the absence of serum, 10 μg/ml of collagen I was added to the lower compartment and cells were added to the upper compartment. The non-migrated cells on the upper side of the chamber were wiped with a cotton swab after 24 h, and the migrated cells on the lower side of the chamber were fixed with 10% ethanol for 25 min, and then stained with 0.4% crystal violet. After rinsing with PBS, three fields of view were randomly selected and counted. Experiments were conducted three times in triplicate.

### 2.8 Cell metabolomic analysis

#### 2.8.1 Sample Preparation

NCI-H1299 cells were seeded in 6-well plates (5× 10^5^ cells/well) and incubated overnight. Then cells were treated with COJE or ZOJE of three concentrations (IC_50_ × 1/2, IC_50_ × 1, IC_50_ × 2), which were set as low (L), medium (M), and high (H) concentration, respectively, for 48 h. At the end of incubation, the cells were rinsed with pre-chilled PBS. Aliquots of the cells were counted with a hemocytometer. Then 1 ml/well of 80% methanol was added to the remaining cells for quenching metabolism and extraction of intracellular metabolites. Subsequently, the cell samples were incubated at −80°C for 30 min, then scraped on the ice and transferred to a 2 ml Eppendorf tube. And 0.4 ml/well of 80% methanol was added into the 6-well plate and the above operations were repeated twice for fully extraction of the metabolites. Then samples were centrifuged at 14,000 rpm under 4°C for 20 min. Following that, 700 μl of each supernatant was retained and dried with nitrogen. The dried samples were reconstituted with 500 μl of acetonitrile/water (6:4) and vortex for 10 min, then centrifugated at 14,000 rpm under 4°C for 20 min. Cell count results in each sample were used to normalize metabolite levels. Pooled “quality control” (QC) samples were prepared to optimize and validate chromatographic and TOF-MS conditions by mixing aliquots of 20 µl of solution from each cell sample.

#### 2.8.2 UHPLC/Q-TOF-MS analysis

Liquid chromatography was conducted by a Waters ACQUITY™ ultra performance liquid chromatography (Waters Corp., Milford, MA, United States). An aliquot (5 μl) of each sample was injected into a Waters ACQUITY UPLC BEH C_18_ column (100 mm × 2.1 mm, 1.7 μm) maintained at 45 °C. The mobile phase consisted of 0.1% formic acid in water (A) and acetonitrile (B) with the following gradient program: 5–30% B (0–1 min), 30–60% B (1–3 min), 60–80% B (3–6 min), 80–90% B (6–8 min), 90–100% B (8–9 min), 100% B (9–11 min), 100–5% B (11–12 min), 5% B (12–15 min). The flow rate was 0.3 ml/min.

The eluent was introduced to a SYNAPT G2Si high-definition mass spectrometer (Waters Corp., Milford, MA, United States) with electrospray ionization (ESI) source to obtain cell metabolic profiles in both positive and negative ion mode. Source temperature was set at 120°C with the cone gas flow of 50 L/h. And the desolvation gas temperature was 500°C with gas flow of 800 L/h. The capillary voltage was set to 3 kV (positive ion mode) or 2.4 kV (negative ion mode), sampling cone voltage was set to 40 V. The TOF acquisition rate was 0.5 s/scan. MS data were collected for all the ions observed in the preceding MS scan. The leucine enkephalin calibration solution (200 pg/ml) was applied to the lock mass in positive ion mode (*m/z* 556.2771) and negative ion mode (*m/z* 554.2615) to ensure the accuracy and reproducibility of Q-TOF-MS. A full scan mass ranges from *m/z* 100 to *m/z* 1000 was scanned.

### 2.9 Data processing and statistical analysis

The raw UHPLC/Q-TOF-MS data were imported to Progenesis QI software (Waters Corporation, MA, United States) for peak detection and alignment to acquire a data matrix containing the *m/z* values, retention time and normalized peak intensity of each sample. The data were subsequently processed according to the metabolomics “80% rule” to filter missing values (Bijlsma et al., 2006) and introduced into the SIMCA software (version 14.1, Umetrics, Umeå, Sweden) for multivariate statistical analysis, involving partial least-squares discrimination analysis (PLS-DA) and orthogonal partial least-squares discrimination analysis (OPLS-DA). Altered metabolites with statistical significance were characterized by variable importance in the projection (VIP) value greater than 1.0 and *p*-value less than 0.05. These putative biomarkers were tentatively identified based on accuracy molecular weight and MS/MS data in available databases such as Human Metabolome database (HMDB, https://www.hmdb.ca/) and Kyoto Encyclopedia of Genes and Genomes (KEGG, https://www.genome.jp/kegg/). Pathway analysis overview was produced by MetaboAnalyst 5.0 (http://www.metaboanalyst.ca/). All data from replicate experiments were expressed as the mean ± standard deviation (SD). Significance was evaluated using Student’s t-test or one-way analysis of variance (ANOVA) using the GraphPad Prism 9.0 (GraphPad Software Inc., San Diego, CA, United States). A probability value of *p* < 0.05 was considered statistically significant.

## 3 Results

### 3.1 Chemical analysis of OJE samples

**FIGURE 1:**
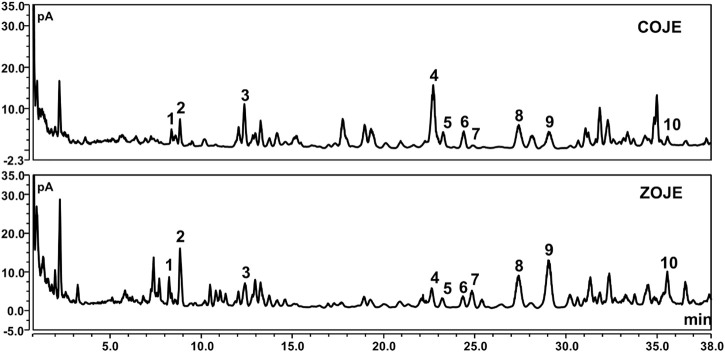
UHPLC-CAD chromatograms of COJE and ZOJE samples. **1**. ophiopogonin C, **2**. ophiopogonin Ra, **3**. 14α-hydroxy-sprengerinin C, **4**. ophiopogonin D, **5**. lirioprolioside J, **6**. ophiopogonin D′, **7**. methylophiopogonone A, **8**. methylophiopogonanone A, **9**. methylophiopogonanone B, **10**.6-aldehydoisoophi opogonanone A.

Six saponins including ophiopogonin C, ophiopogonin Ra, 14α-hydroxy-sprengerinin C, ophiopogonin D, lirioprolioside J and ophiopogonin D’, as well as four homoisoflavanoids involving methylophiopogonone A, methylophiopogonanone A, methylophiopogonanomne B and 6-aldehydoisoophiopogonanone A, were quantitatively determined in OJE samples by UHPLC. CAD, a near-universal detector, was chosen due to the lack of chromophores of the saponins. As presented in Table 1 and Figure 1, the contents of individual saponins were different in COJE and ZOJE, although the total contents of the six saponins were similar, i.e., 2.81 mg/g in COJE and 2.48 mg/g in ZOJE, respectively. While ZOJE contained higher contents of homoisoflavonoids than COJE, which were 4.53 mg/g in ZOJE and 1.75 mg/g in COJE, respectively.

**TABLE 1:** Contents (mg/g) of six saponins and four homoisoflavonoids in OJE (n = 3, mean ± SD).

No	Analytes	Contents in COJE	Contents in ZOJE
1	Ophiopogonin C	0.18 ± 0.00	0.37 ± 0.00
2	Ophiopogonin Ra	0.39 ± 0.01	1.04 ± 0.03
3	14α-Hydroxy-sprengerinin C	0.51 ± 0.01	0.38 ± 0.01
4	Ophiopogonin D	1.08 ± 0.03	0.27 ± 0.01
5	Lirioprolioside J	0.23 ± 0.00	0.14 ± 0.00
6	Ophiopogonin D′	0.42 ± 0.01	0.28 ± 0.01
7	Methylophiopogonone A	0.08 ± 0.00	0.40 ± 0.01
8	Methylophiopogonanone A	0.76 ± 0.00	1.21 ± 0.03
9	Methylophiopogonanone B	0.77 ± 0.03	2.20 ± 0.05
10	6-Aldehydoisoophiopogonanone A	0.14 ± 0.00	0.72 ± 0.01

### 3.2 OJE inhibits the viabilities of human lung cancer cells

**FIGURE 2:**
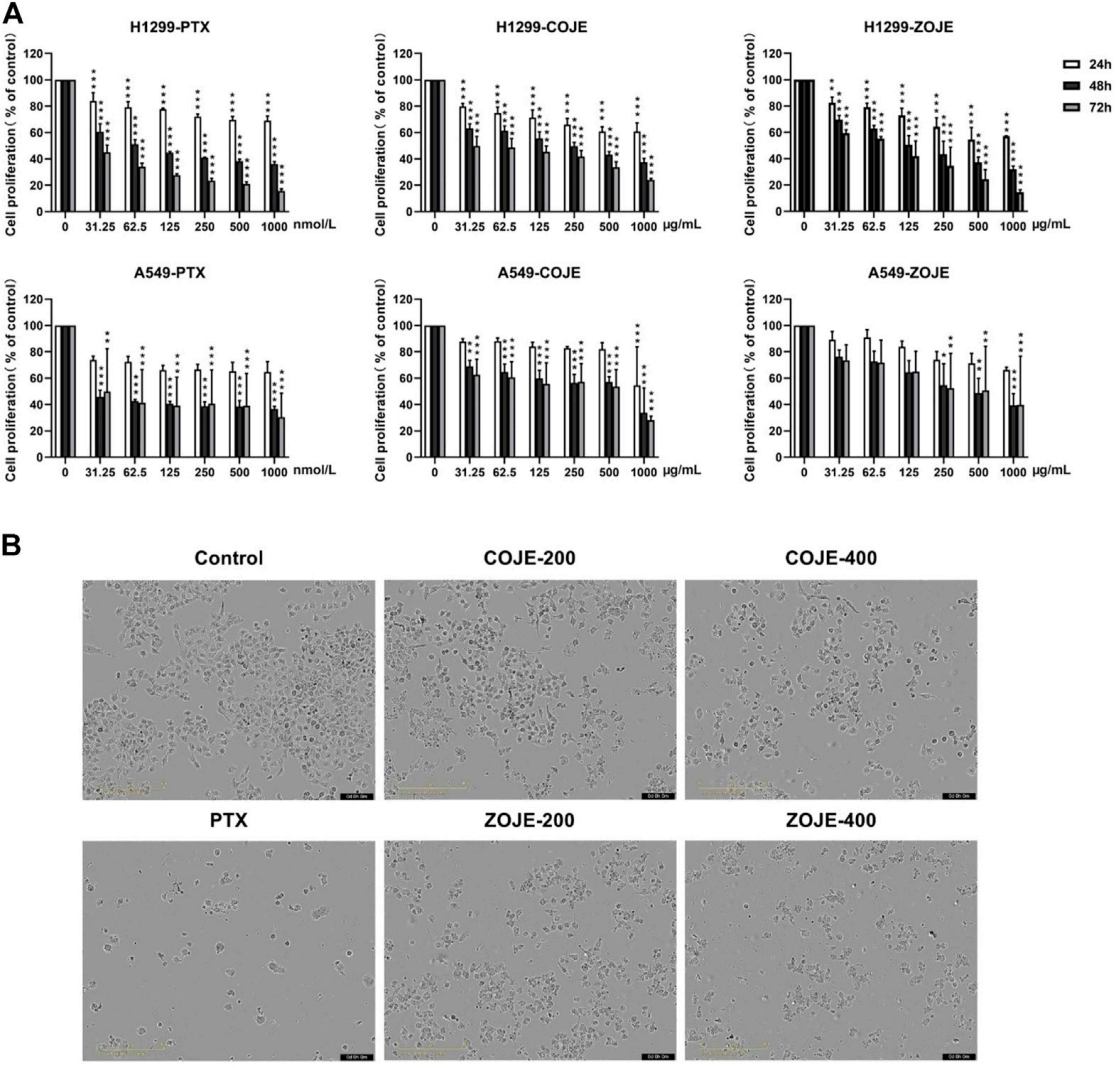
Cell proliferation of NCI-H1299 and A549 cells after OJE treatment. (**A**) NCI-H1299 and A549 cells were exposed to OJE (0–1000 μg/ml) or PTX (0–1000 nmol/L) for 24–72 h, and cell proliferation was determined utilizing MTT assay. (**B**) Morphological changes of NCI-H1299 cells after treatment with vehicle, OJE (200, and 400 μg/ml) or PTX (500 nmol/L) for 48 h. Scale bar: 100 μm. The experimental data are expressed as the mean value with SD of three independent replicates. (*p < 0.05, **p < 0.01, ***p < 0.001, compared to control).

Firstly, to evaluate the cytotoxicity of OJE, MTT assay was conducted in two lung cancer cell lines (NCI-H1299 and A549). As shown in Figure 2A, PTX had a significantly inhibition of NCI-H1299 and A549 cells proliferation in a time-dependent manner as previous report (Georgiadis et al., 1997). Moreover, COJE and ZOJE significantly inhibited the viability of NCI-H1299 and A549 cells in a time- and concentration-dependent manner. The IC_50_ of COJE and ZOJE on NCI-H1299 cells at 48 h were calculated to be 259.5 ± 40.9 μg/ml and 140.6 ± 12.3 μg/ml, respectively. While those on A549 cells were 330.6 ± 45.5 μg/ml and 411.8 ± 66.5 μg/ml, respectively. Therefore, NCI-H1299 cells were chosen for further investigation. The morphological changes of NCI-H1299 cells after OJE-treatment for 48 h also showed that OJE induced obvious cell death in NCI-H1299 cells (Figure 2B).

### 3.3 OJE induces apoptosis and inhibits migration

**FIGURE 3:**
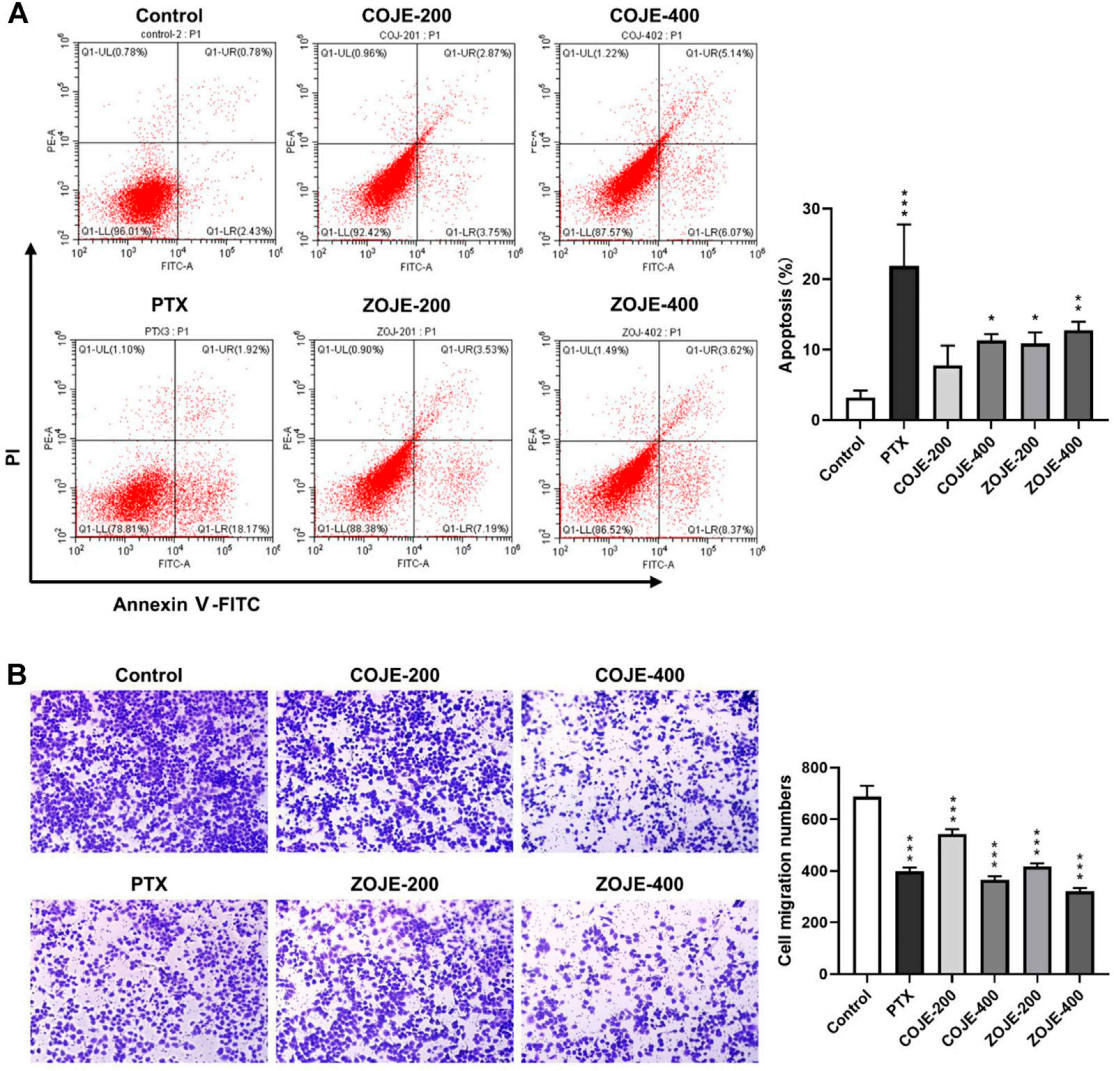
OJE induced apoptosis and inhibited migration of NCI-H1299 cells. (**A**) Induction of apoptosis in NCI-H1299 cells treated with vehicle, OJE (200, and 400 μg/ml) or PTX (500 nmol/L). NCI-H1299 cells were exposed to vehicle, OJE or PTX at designated concentrations for 48 h. The cell apoptosis rate was treated statistically. (**B**) NCI-H1299 cells were seeded in the top chamber of transwell with serum-free medium and treated with vehicle, OJE (200, and 400 μg/ml) or PTX (500 nmol/L). After about 48 h, migrated cells were fixed, stained, photographed and quantified (10×). Migrated cells in three fields selected randomly were counted and photographed under a light microscope. Data are expressed as mean ± SD of three independent replicates. (*p < 0.05, **p < 0.01, ***p < 0.001 compared with control).

The flow cytometry assay was carried out using Annexin V/PI double staining to determine the cell apoptosis. Treatment with PTX (500 nmol/L) for 48 h significantly increased the apoptosis rate, while COJE or ZOJE (200, and 400 μg/ml) induced slight apoptosis in NCI-H1299 cells in concentration-dependent manners (Figure 3A). To investigate the potential effects of OJE on lung cancer metastasis, transwell assay was performed on NCI-H1299 cells. The results showed that the cell migration capability was significantly inhibited by COJE and ZOJE in a concentration-dependent manner (Figure 3B).

### 3.4 UHPLC/Q-TOF-MS method validation

To assess the reproducibility and stability of the UHPLC/Q-TOFMS method, 20 μl from each sample were mixed to obtain the QC sample. A pooled QC sample containing all metabolites was injected into the instrument 3–5 times at the beginning of sequence, and analyzed once or twice every eight samples. The reproducibility of the metabolomics analysis was evaluated by relative standard deviation (RSD%) after alignment and normalization. A total of 80% of the variables among 2548 ions acquired from the QC sample had RSD% less than 20% in ESI positive ion mode, and 82% of variables among the 2989 ions acquired from the QC sample had RSD% less than 20% in ESI negative ion. These results demonstrated that the developed method could be used for sequence analysis of cell metabolomic samples with good repeatability and stability.

### 3.5 Metabolomics analysis of NCI-H1299 cells treated by OJE

**FIGURE 4:**
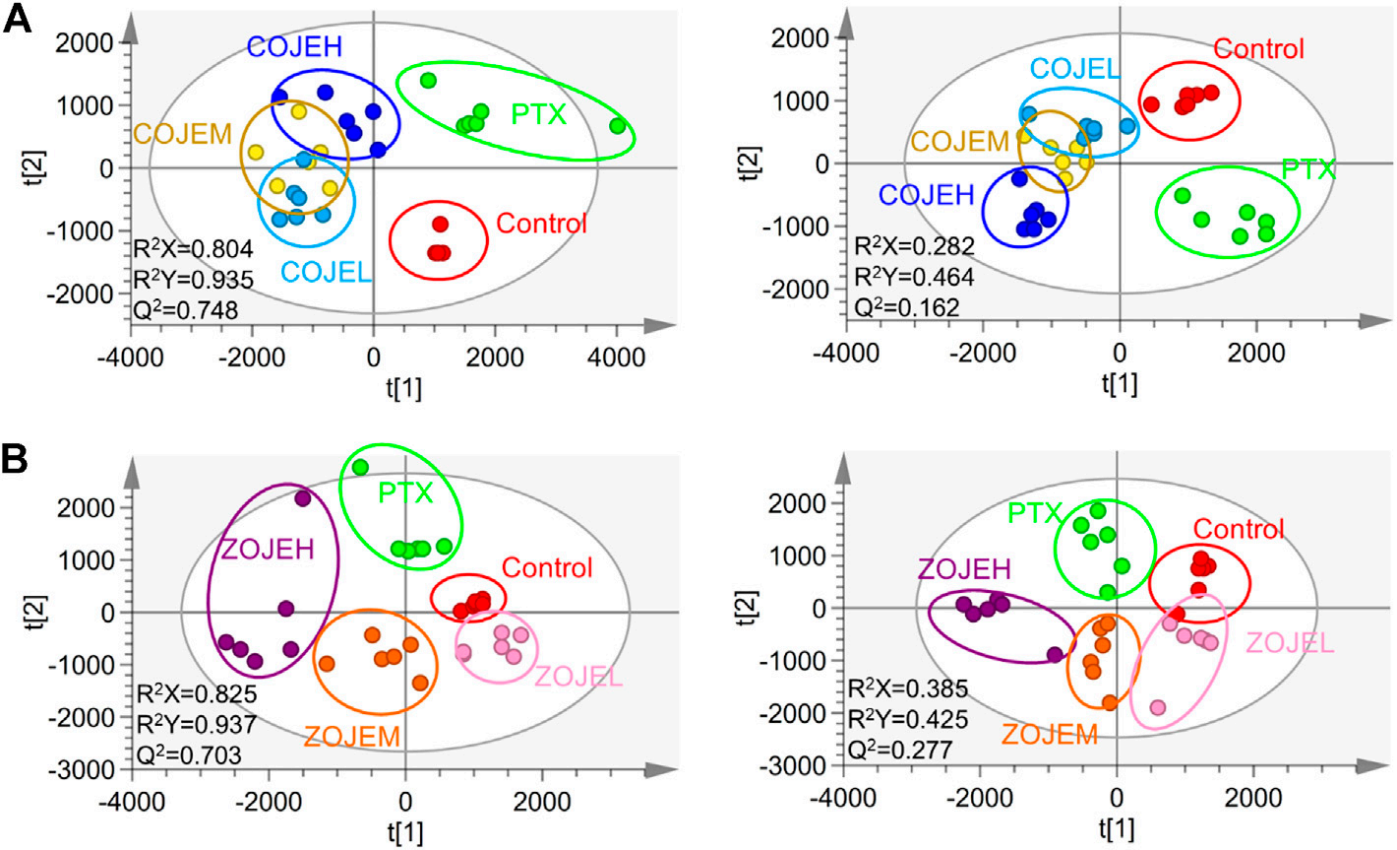
PLS-DA score plot based on the UHPLC/Q-TOF-MS spectra of NCI-H1299 cell samples in control, PTX and different concentration of **(A)** COJE- or **(B)** ZOJE-treated groups detected in positive ion mode (left) and negative ion mode (right).

The data matrix containing the *m/z* values, retention time, and normalized peak intensity of each sample was imported into the SIMCA software for multivariate statistical analysis. A supervised PLS-DA model was applied to evaluate the intervention effects of various concentrations of OJE on the NCI-H1299 cells. As shown in Figure 4A, based on the metabolic profiles in positive ion mode, the COJE groups were further away from the control group and approached the PTX group with the increase of the concentration, which showed good reversal trends. The R^2^Y and Q^2^ (cum) values were 0.935 and 0.748, respectively, indicating good fitness and prediction of the constructed PLS-DA model. While in negative ion mode, although the R^2^Y and Q^2^ (cum) values of 0.464 and 0.162 were relatively low, the same trends in the score plot could be observed. Similarly, ZOJE groups also showed good reversal trends in a concentration-dependent manner (Figure 4B). These results suggested metabolic profiles could reflect the anticancer effects of OJE against NCI-H1299 cells.

### 3.6 Discovery and identification of potential biomarkers

**FIGURE 5:**
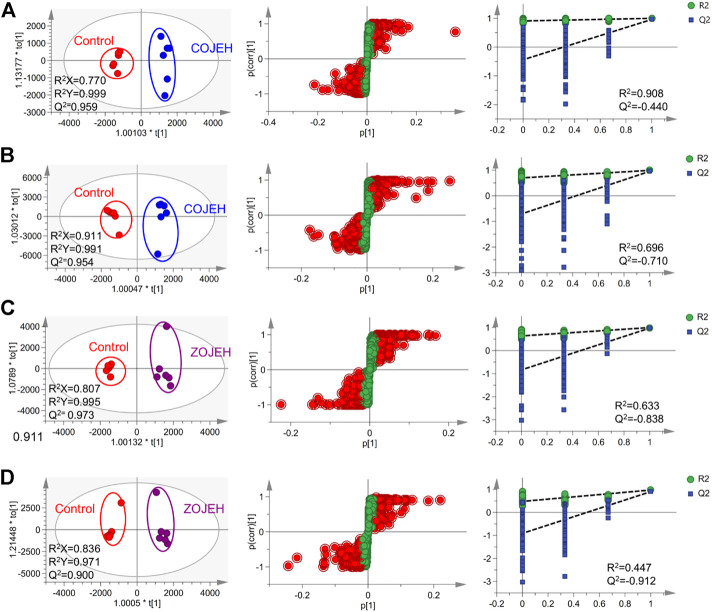
OPLS-DA score plot (left), S-plot (middle) and permutations test (right) based on the UHPLC/Q-TOF-MS spectra of NCI-H1299 cell samples in control and (**A,B**) COJEH- or (**C,D**) ZOJEH- treated group detected in (**A,C**) positive ion mode and (**B,D**) negative ion mode. Potential biomarkers with VIP >1.0 were marked as red.

To further identify potential biomarkers, OPLS-DA model was used to distinguish the characteristics of control group and high-concentration of COJE (COJEH) or ZOJE (ZOJEH) group in positive and negative ion mode, respectively. All the R^2^Y and Q^2^ (cum) values were larger than 0.9 and close to 1.0 (Figure 5), suggesting the OPLS-DA models had excellent fitness and strong predictability. The permutation tests (200 iterations) of the OPLS-DA results showed that the models were stable and reliable without overfitting (Figure 5). Subsequently, variables based on VIP values (>1.0) were further filtered using Student’s t-test to assess whether these potential differential metabolites were statistically significant between control and COJEH/ZOJEH groups. Features with VIP >1.0 and *p* < 0.05 of differential metabolites were considered as potential biomarkers that had a remarkable contribution to the discrimination between the control and COJEH/ZOJEH groups. Potential biomarkers were tentatively identified according to molecular weight and MS/MS spectra. The exact molecular formulas were searched from the biochemical database based on the accurate molecular mass acquired from the robust Q-TOF-MS analysis platform. Then the structural characteristics of biomarkers were obtained by comparing their MS/MS data associated with the corresponding product ion fragment. Finally, a total of 22 potential biomarkers contributing to the anticancer effects of OJE in NCI-H1299 cells were tentatively assigned and the results were given in Table 2.

**TABLE 2:** Potential biomarkers associated with the anticancer effect of PTX and OJE in NCI-H1299 cells.

No	Identified metabolites	Retention time (min)	*m/z*	Adduct ions	Mass error (ppm)	PTX vs control		COJEH vs control		ZOJEH vs control		Metabolic pathways
						VIP	Trend	VIP	Trend	VIP	Trend	
1	Glycerophosphocholine	0.83	258.1104	[M + H]^+^	1.18	0.32	-	1.75	↓	2.05	↓	Glycerophospholipid metabolism, Ether lipid metabolism
2	NADH	0.89	666.1302	[M + H]^+^	−0.04	1.99	↓	1.27	-	1.87	↓	Oxidative phosphorylation
3	Oxidized glutathione	0.90	613.1599	[M + H]^+^	−7.40	4.58	↓	2.37	-	3.82	↓	Glutathione metabolism
4	Glutathione	0.92	308.0913	[M + H]^+^	2.52	14.46	↓	8.05	↓	12.28	↓	Glutathione metabolism
5	Butyrylcarnitine	1.66	232.1542	[M + H]^+^	−0.49	1.37	↓	0.70	↓	1.14	↓	Unknown
6	Isovalerylcarnitine	1.87	246.1699	[M + H]^+^	−0.40	2.18	↓	2.26	↓	2.29	↓	Unknown
7	Sphingosine	4.85	282.2791	[M + H-H_2_O]^+^	−0.27	1.34	-	1.68	↑	1.34	↓	Sphingolipid metabolism
8	LysoPE (20:4)	5.26	500.2775	[M-H]^-^	−1.65	1.99	↑	1.26	↑	1.20	↑	Glycerophospholipid metabolism
9	LysoPE (18:2)	5.28	476.2774	[M-H]^-^	−1.77	0.30	-	1.51	↑	0.85	↑	Glycerophospholipid metabolism
10	LysoPC(20:4)	5.29	544.3394	[M + H]^+^	3.96	1.25	↑	1.50	↑	1.13	↑	Glycerophospholipid metabolism
11	LysoPC(22:5)	5.50	614.3448	[M + FA-H]^-^	−2.67	1.53	↑	1.37	↑	1.35	↑	Glycerophospholipid metabolism
12	LysoPC(20:3)	5.71	546.3536	[M + H]^+^	−3.37	0.56	-	1.56	↑	0.90	↑	Glycerophospholipid metabolism
13	LysoPE (16:0)	5.77	452.2774	[M-H]^-^	−1.94	3.42	↓	0.42	↓	2.68	-	Glycerophospholipid metabolism
14	LysoPE (18:1)	6.05	478.2933	[M-H]^-^	−1.25	3.43	↓	0.71	↓	2.71	↓	Glycerophospholipid metabolism
15	PE (P-16:0)	6.10	436.2822	[M-H]^-^	−2.72	1.71	↓	0.43	↓	1.56	-	Ether lipid metabolism
16	PC(P-16:0)	6.13	480.3428	[M + H]^+^	−4.28	1.32	↓	1.14	-	1.11	↓	Ether lipid metabolism
17	1-Hexadecyl lysophosphatidylcholine	6.14	482.3602	[M + H]^+^	−0.52	3.22	↓	2.17	↓	2.48	↓	Unknown
18	LysoPC(20:2)	6.38	548.3688	[M + H]^+^	-4.04	0.32	-	1.54	↑	0.26	-	Glycerophospholipid metabolism
19	LysoPA (18:0)	6.84	483.2713	[M + FA-H]^-^	−3.43	1.53	↑	2.27	↑	1.75	↑	Glycerolipid metabolism Glycerophospholipid metabolism
20	PS(18:0)	7.74	524.2980	[M-H]^-^	−2.62	1.23	-	3.34	-	1.29	↑	Glycerophospholipid metabolism
21	Calcidiol	8.52	383.3307	[M + H-H_2_O]^+^	−0.37	2.37	-	3.46	↑	1.27	-	Steroid biosynthesis
22	Bis(2-ethylhexyl) phthalate	10.08	413.2666	[M + Na]^+^	0.95	5.26	-	5.81	-	5.54	↑	Unknown

**FIGURE 6:**
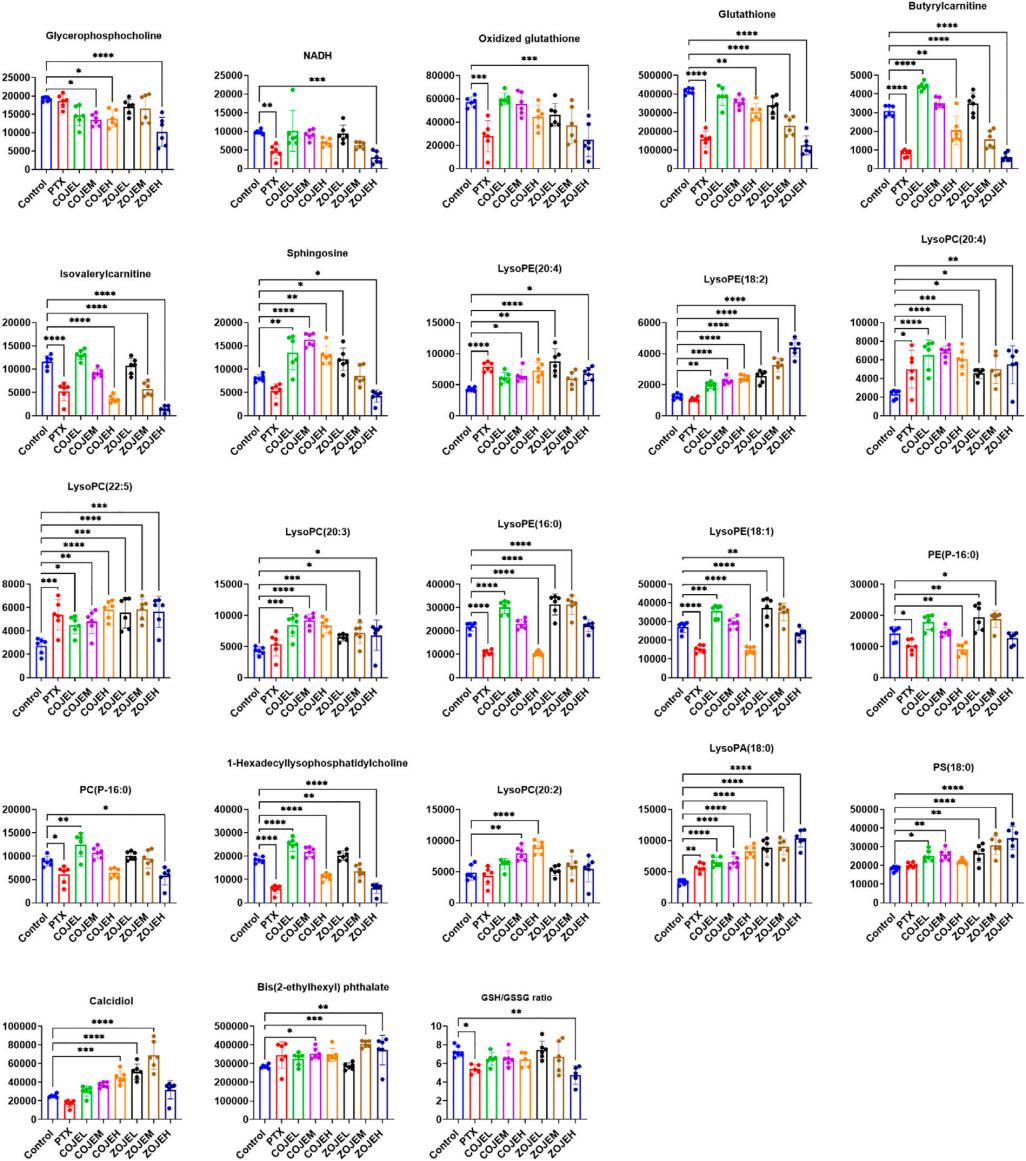
Changes in the relative peak intensities of 22 identified metabolites and the ratio of GSH/GSSG in different groups. (GSH, glutathione; GSSG, oxidized glutathione; *p < 0.05, **p < 0.01, ***p < 0.001, ****p < 0.0001, compared with control group).

The relative peak intensities of the 22 identified potential biomarkers in each group were shown in Figure 6. Compared with the control group, 10 metabolites including NADH, oxidized glutathione, glutathione, butyrylcarnitine, isovalerylcarnitine, lysoPE (16:0), lysoPE (18:1), PE (P-16:0), PC(P-16:0) and 1-hexadecyllysophosphatidylcholine were decreased significantly in the PTX group, and they were also down-regulated in the COJEH and/or ZOJEH groups. Four metabolites involving lysoPE (20:4), lysoPC(20:4), lysoPC(22:5) and lysoPA (18:0) were increased remarkably in the PTX group, and they were also up-regulated in the COJEH and ZOJEH groups. Glycerophosphorylcholine had no significant difference in the PTX group, but it was decreased in the COJEH and ZOJEH group. The other seven metabolites including sphingosine, lysoPE (18:2), lysoPC(20:3), lysoPC(20:2), PS(18:0), calcidiol and bis(2-ethylhexyl) phthalate did not change obviously in PTX group, but they were increased in COJEH and/or ZOJEH groups.

### 3.7 Metabolic pathway analysis

**FIGURE 7:**
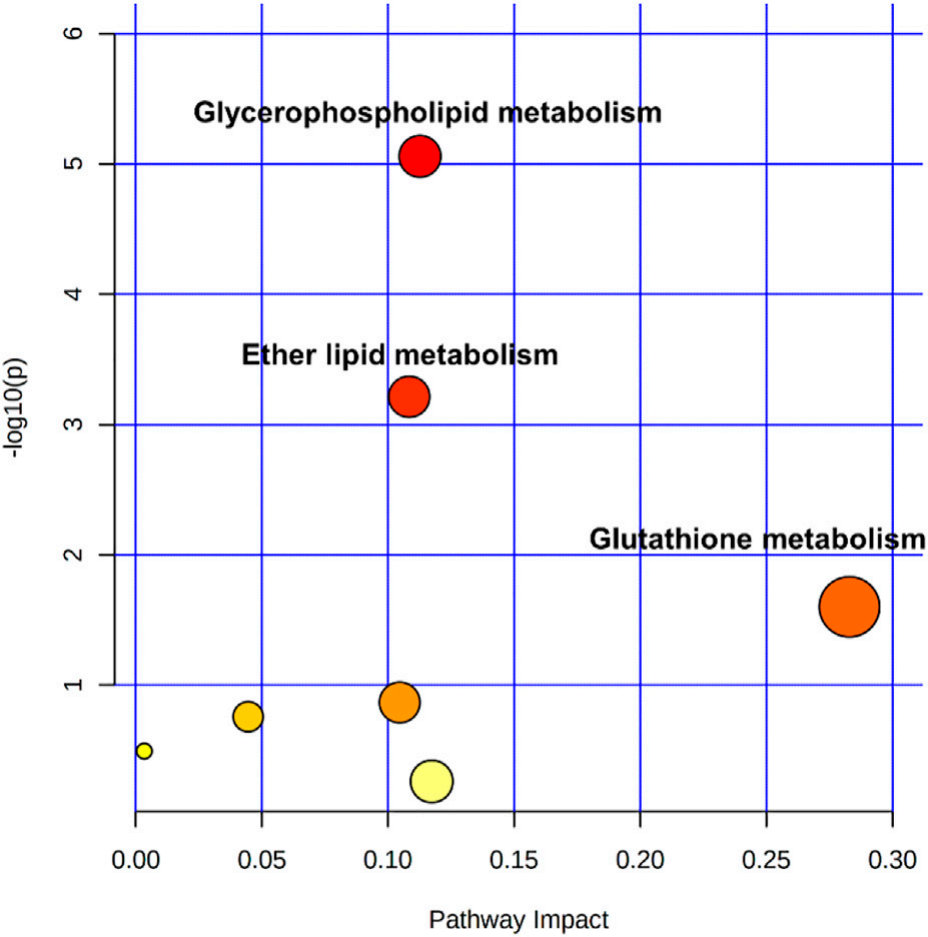
Pathway analysis overview depicting altered metabolic pathways in NCI-H1299 cells from control and COJEH/ZOJEH-treated groups. The metabolic pathways are displayed as distinctly colored circles depending on their enrichment analysis scores (vertical axis, shade of red) and topology (pathway impact, horizontal axis, circle diameter) via MetaboAnalyst 5.0.

These potential biomarkers were imported into MetaboAnalyst 5.0 for metabolic pathways analysis. As shown in pathway analysis bubble plot (Figure 7), seven metabolic pathways were affected in the OJEH-treated groups compared with the control group. Among them, glycerophospholipid metabolism, ether lipid metabolism, and glutathione metabolism were considered as significantly changed pathways with impact values greater than 0.10 and -log *p* values more than 1.3 (namely *p* < 0.05).

## 4 Discussion

Previous reports show that COJ and ZOJ contain similar types of steroidal saponins and homoisoflavonoids, but their composition and concentrations are different (Lin et al., 2010; Zhao M. et al., 2017). In this study, chemical analysis showed that the total contents of the six saponins were similar in COJE and ZOJE, but the contents of individual saponins were different. In addition, ZOJE contained higher contents of homoisoflavonoids than COJE (Table 1 and Figure 1). These results are consistent with previous reports (Lin et al., 2010; Zhao M. et al., 2017). Differences in the chemical composition may lead to different pharmacological effects of OJ from different producing regions. Lu et al. found that ZOJ might possess better immunomodulatory activities than COJ (Lu et al., 2017). Zhao et al. reported that ZOJ possessed stronger antioxidant and anti-inflammatory abilities than COJ and they exhibited selective cytotoxicity on different cancer cell lines *in vitro* (Zhao M. et al., 2017). These findings suggested that OJ from different regions should be evaluated and considered in clinical application. In present study, MTT assays results showed that OJE was more sensitive to NCI-H1299 cell viability, at the IC_50_ of 259.5 ± 40.9 μg/ml (COJE) and 140.6 ± 12.3 μg/ml (ZOJE), respectively. COJE and ZOJE induced mild apoptosis in NCI-H1299 cells, and significantly inhibited cell migration in a concentration-dependent manner. Moreover, the pro-apoptotic and anti-migratory abilities of ZOJE on NCI-H1299 were more significant than those of COJE as shown in Figure 3, which may be due to their different chemical compositions. It was speculated that the better antioxidative and anti-inflammatory efficacies of ZOJ might be owing to the higher contents of homoisoflavonoids in ZOJ than those in COJ, and the diverse anticancer capacities in different cell lines of COJ and ZOJ might result from the unique steroidal saponins in the two OJ (Zhao M. et al., 2017). However, these speculations have not been verified, and the relationships between the chemical components and the bioactivities of OJ are still not clear, which needs further exploration in the future.

**FIGURE 8:**
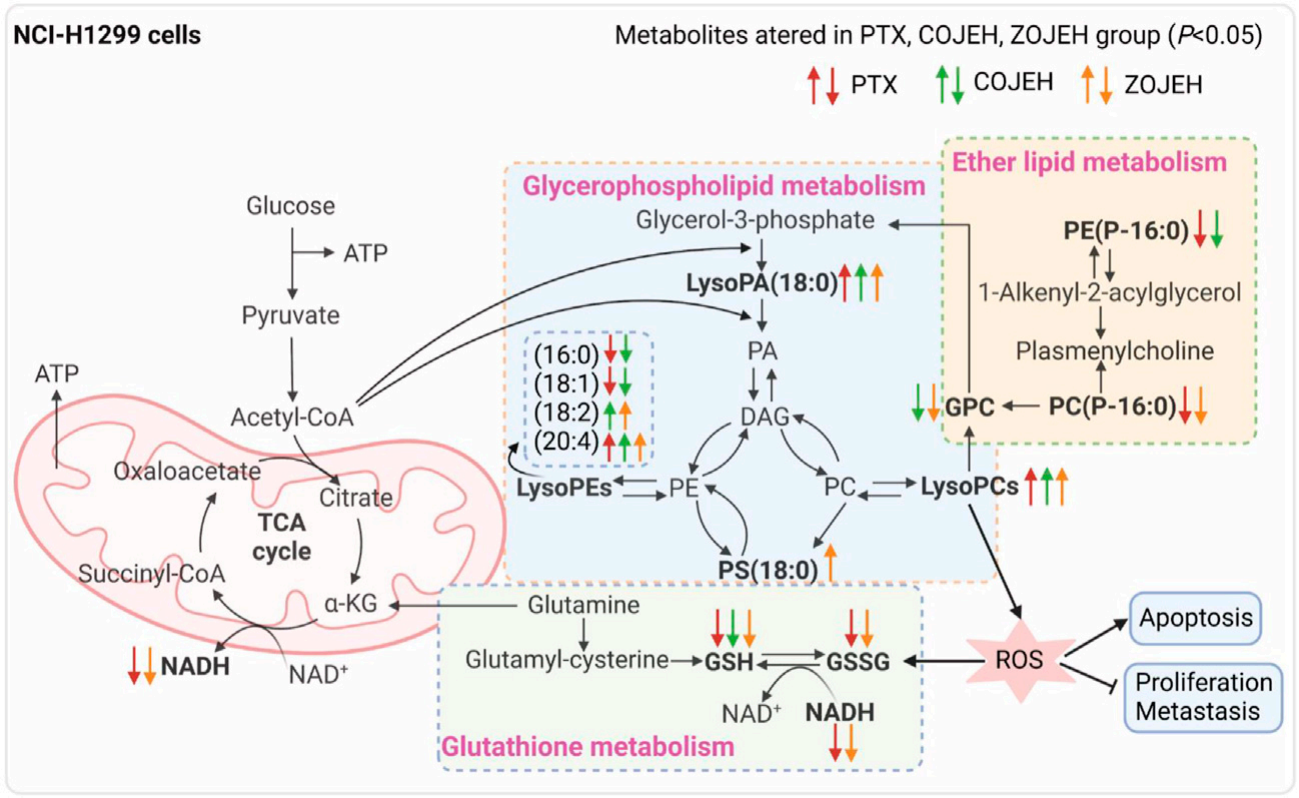
Significantly altered metabolic pathways. Metabolites in bold are metabolites disturbed by PTX, COJEH and/or ZOJEH group. Red, green and orange arrows represent up-regulation/down-regulation by PTX, COJEH and ZOJH group, respectively, when compared with that in control group. (PA, phosphatidic acid; DAG, diacylglycerol; PC, phosphatidylcholine; PE, phosphatidylethanolamine; PS, phosphatidylserine; GPC, glycerophosphocholine; α-KG, alpha ketoglutarate; GSH, glutathione; GSSG, oxidized glutathione; ROS, reactive oxygen species.) The image was created with BioRender.com.

To further elucidate the anticancer mechanisms from a metabolic perspective, UHPLC/Q-TOF-MS-based metabolomics approach was applied to the analysis of NCI-H1299 cells treated with COJE or ZOJE (low, medium, and high concentration) for 48 h. Compared with the control group, the metabolites detected in both positive and negative ion modes changed greatly and showed good reversal trends in concentration-dependent manners as shown in Figure 4. A total of 22 biomarkers were found to be possibly related to the different properties of the control group and COJEH/ZOJEH group, among which glycerophospholipid metabolism, ether lipid metabolism and glutathione metabolism were highlighted as the significantly disturbed pathways (Figure 8). In addition, the relative peak intensities of the 22 biomarkers in different groups showed the changing trends of metabolite levels (Figure 6). The contents of glycerophosphocholine, NADH, oxidized glutathione, glutathione, butyrylcarnitine, isovalerylcarnitine, lysoPE (18:2), PC(P-16:0), 1-hexadecyllysophosphatidylcholine, lysoPA (18:0) and PS(18:0) in the control group were reversed more significantly in the ZOJE group. While the levels of lysoPC(20:3), lysoPE (16:0), lysoPE (18:1), PE (P-16:0) and lysoPC(20:2) in the control group were reversed more remarkably in the COJE group. The different changes in the above metabolites may be the important factors for the difference in the anticancer effects of COJE and ZOJE.

Abnormal lipid metabolism is closely related to carcinogenesis and cancer metastasis. The malignant transformation and accelerated proliferation of tumor cells have a high demand for energy, which can cause changes in lipid metabolism to supply the formation of various organelles and their own special physiological needs (Pavlova and Thompson, 2016; Long et al., 2018). Previous study has indicated that glycerophospholipid metabolism is the most significantly altered metabolic pathway in lung cancer patients (Chen Y. et al., 2018). As the intermediate of PCs and PEs, lysoPCs and lysoPEs have been considered as biomarkers of many diseases. They can regulate a variety of biological processes, including cell proliferation, tumor cell invasion and inflammation (Raynor et al., 2015; Ross et al., 2016). After exposure to COJE or ZOJE, lysoPE (18:2), lysoPE (20:4), lysoPC(20:2), lysoPC(20:3), lysoPC(20:4), lysoPC(22:5), lysoPA (18:0), and PS(18:0) were significantly up-regulated, while glycerophosphocholine, lysoPE (16:0) and lysoPE (18:1) were down-regulated, suggesting that OJE may disturb the proliferation of tumor cells and the stability of cell membrane structure via the regulation of the activities of enzymes involved in the biosynthesis of glycerophospholipids, such as secretory phospholipase A2 (SPLA2), lecithin-cholesterol acyltransferase (LCAT), and lysophospholipid acyltransferase (LPCAT4) (Shindou et al., 2009). In addition, the membrane fluidity and signal transduction of tumor cells may be affected by changes in lipids, thus playing an important role in tumor metastasis and development. Down-regulation of ether lipids [PE (P-16:0) and PC(P-16:0)] is closely associated with abnormally high levels of oxidative stress and peroxisomal dysfunction in lung cancer (Yang et al., 2020).

Glutathione is the main intracellular antioxidant buffer against oxidative stress, mainly in the form of reduced glutathione (GSH) and oxidized glutathione (GSSG) (Lv et al., 2019). Under physiological conditions, the reduced form of GSH is the predominant form and its concentration is 10 to 100-fold higher than that of the oxidized form (GSSG). Under oxidative stress, GSH is converted to GSSG by GSH-dependent peroxidase after reaction with reactive oxygen species (ROS) (Bansal and Simon, 2018). The ratio of GSH and GSSG provides insight into the extent of glutathione against oxidative stress and is often used as a marker of cytotoxicity. Studies have demonstrated the importance of GSH and GSH/GSSG ratios in regulating tumor cell viability, tumor development, progression, and initiation of drug resistance (Huang et al., 2001; Kalinina and Gavriliuk, 2020). Furthermore, glutathione is an important signaling controller of cellular differentiation, proliferation, apoptosis, ferroptosis and immune function (Kennedy et al., 2020). In this study, GSH was down-regulated after treatment by COJE or ZOJE in a concentration-dependent manner, and GSH/GSSG ratio was significant down-regulated after treatment by PTX or ZOJEH groups (Figure 6). The high production of ROS in cancer cells requires a highly active antioxidant system in the cell, so reducing glutathione production or availability provides a pathway for cancer therapy. Furthermore, depletion of GSH in cancer cells can ultimately lead to ferroptosis (Li et al., 2020; Kang et al., 2021).

## 5 Conclusion

In this study, COJE- and ZOJE-induced metabolite changes in NCI-H1299 cells were analyzed using UHPLC/Q-TOF-MS-based cell metabolomics. The significantly altered metabolites were involved in glycerophospholipid metabolism, ether lipid metabolism and glutathione metabolism. This study helps to understand the intramolecular metabolic process of NCI-H1299 cells induced by COJE and ZOJE, and may provide clues for understanding their potential anticancer mechanism. In the future, further *in vivo* experiments are necessary to evaluate the specific relationship between pharmacological effects and metabolite alterations to elucidate the anticancer mechanism of OJ.

## Data Availability

The raw data supporting the conclusions of this article will be made available by the authors, without undue reservation.
